# Activity-Based Anorexia Alters the Expression of BDNF Transcripts in the Mesocorticolimbic Reward Circuit

**DOI:** 10.1371/journal.pone.0166756

**Published:** 2016-11-18

**Authors:** Emily V. Ho, Stephanie J. Klenotich, Matthew S. McMurray, Stephanie C Dulawa

**Affiliations:** 1 Department of Psychiatry, University of California San Diego, La Jolla, California, United States of America; 2 Department of Psychiatry, University of Chicago, Chicago, Illinois, United States of America; 3 Department of Psychology, University of Illinois Chicago, Chicago, Illinois, United States of America; Kent State University, UNITED STATES

## Abstract

Anorexia nervosa (AN) is a complex eating disorder with severe dysregulation of appetitive behavior. The activity-based anorexia (ABA) paradigm is an animal model in which rodents exposed to both running wheels and scheduled feeding develop aspects of AN including paradoxical hypophagia, dramatic weight loss, and hyperactivity, while animals exposed to only one condition maintain normal body weight. Brain-derived neurotrophic factor (BDNF), an activity-dependent modulator of neuronal plasticity, is reduced in the serum of AN patients, and is a known regulator of feeding and weight maintenance. We assessed the effects of scheduled feeding, running wheel access, or both on the expression of BDNF transcripts within the mesocorticolimbic pathway. We also assessed the expression of neuronal cell adhesion molecule 1 (NCAM1) to explore the specificity of effects on BDNF within the mesocorticolimbic pathway. Scheduled feeding increased the levels of both transcripts in the hippocampus (HPC), increased NCAM1 mRNA expression in the ventral tegmental area (VTA), and decreased BDNF mRNA levels in the medial prefrontal cortex (mPFC). In addition, wheel running increased BDNF mRNA expression in the VTA. No changes in either transcript were observed in the nucleus accumbens (NAc). Furthermore, no changes in either transcript were induced by the combined scheduled feeding and wheel access condition. These data indicate that scheduled feeding or wheel running alter BDNF and NCAM1 expression levels in specific regions of the mesocorticolimbic pathway. These findings contribute to our current knowledge of the molecular alterations induced by ABA and may help elucidate possible mechanisms of AN pathology.

## Introduction

Brain derived neurotrophic factor (BDNF) is the most abundant neurotrophin in the brain [1], and has been implicated in a number of psychiatric disorders [2]. BDNF is involved in neuroplasticity, the homeostatic regulation of food intake and energy expenditure, stress responsivity, and reward processing [3,4,5,6]. Therefore, BDNF has been hypothesized to be an important molecule in the development and maintenance of anorexia nervosa (AN) [7], an eating disorder characterized by hypophagia, weight loss, hyperactivity, and an intense fear of weight gain.

BDNF expression levels influence behaviors that are relevant to AN, such as feeding and exercise, and are also affected by these behaviors. A meta-analysis of studies assessing serum BDNF concentrations in AN indicates that levels are significantly decreased in patients versus controls [8]. Weight-recovered AN patients show normalization, or even small increases, in serum BDNF concentrations [9]. In contrast, animal studies have shown that BDNF heterozygous (HT) mice exhibit hyperphagia, obesity, and increased locomotor activity [10], while knockout (KO) of BDNF is lethal [11]. Furthermore, dietary restriction normalizes these behaviors, and increases BDNF levels to normal in BDNF HT mice [12]. Additionally, central infusion of BDNF leads to severe appetite suppression and weight loss in rats [13]. Although some small-scale human genetic studies have implicated genetic variants of BDNF in the pathogenesis of AN [14,15], large-scale genome-wide association studies (GWAS) of AN have not identified BDNF as a source of risk [16]. Therefore, the relationship between BDNF levels, feeding, and exercise is complex and the role of BDNF in anorexic behaviors is currently unclear.

The mesocorticolimbic reward circuit, which includes projections from the ventral tegmental area (VTA) to the medial prefrontal cortex (mPFC), hippocampus (HPC), and nucleus accumbens (NAc), modulates addictive behaviors [17], mood [18], and feeding behaviors [19], all of which are altered in AN. AN patients exhibit alterations in reward processing [20,21,22,23], increased anhedonia [24], anxiety [25], and exercise addiction [26]. Neuroimaging studies have revealed altered activation patterns within mesocorticolimbic regions in AN patients [27,28,23].

The activity-based anorexia (ABA) paradigm provides a model for the hypophagia, hyperactivity, and weight loss observed in AN. In the ABA paradigm, rodents simultaneously exposed to running wheels and scheduled feeding rapidly develop hypophagia, weight loss, and paradoxical increases in wheel running [29,30]. Progression of ABA is characterized by hypothermia, loss of estrus, increased HPA axis activity, and ultimately stomach ulceration and death [31,32,29,30,33]. ABA has also been suggested to model exercise addition, since the opioid antagonist naloxone precipitates withdrawal in animals in the ABA paradigm [34]. Furthermore, the excessive exercise that AN patients often engage in has been suggested to qualify as exercise addiction (Klein, 2004). The choice between the rewarding or aversive aspects of wheel running versus food intake is likely influenced by the state of the mesocorticolimbic reward circuit.

We investigated the effects of ABA on BDNF mRNA expression within the VTA, nucleus accumbens, medial prefrontal cortex, and hippocampus using quantitative PCR. We examined gene expression in mice exposed to four conditions: *ad libitum* feeding (Ad Lib), scheduled feeding (or starved mice, STV), free access to running wheels and ad libitum feeding (RUN), and free access to running wheels and scheduled food access (ABA). We also measured the expression of neuronal cell adhesion molecule 1 (NCAM1) mRNA, a recognition molecule important for the formation and modulation of synaptic contacts [35], to explore the specificity of effects on BDNF in these regions. Results should inform future research by identifying neurobiological substrates that correlate with anorexic-like behaviors.

## Materials and Methods

### Animals

Experimentally naïve Balb/cJ female mice (Jackson Laboratories, Bar Harbor, Maine, USA) were aged 9 weeks at the beginning of experimental procedures. Mice received ad libitum access to standard chow and water, except during scheduled feeding. All procedures were conducted in accord with the National Institutes of Health laboratory animal care guidelines and with the Institutional Animal Care and Use Committee approval at the University of Chicago.

### Experimental conditions

Mice were housed in a climate-controlled room maintained on a 12:12 light-dark cycle (lights off at 2000 hours). Cages (19.56 x 34.70 x 14.41 cm) were equipped with wireless low profile running wheels (Med Associates Inc., St. Albans, Vermont, USA). Running wheels transmitted running data every 30 seconds to a computer with Wheel Manager Software 24 hours a day. Food was provided in a glass jar (65 cm diameter x 50 cm height) during baseline and restriction periods.

### Activity-based anorexia paradigm

**Fig 1:**
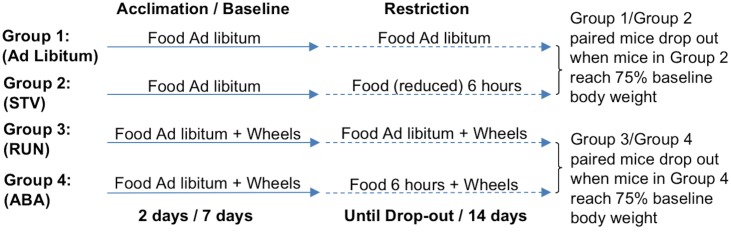
ABA paradigm experimental design.

All animals were pseudo-randomly divided into four experimental groups—Ad Lib, STV, RUN, or ABA—based on body weight upon arrival. During acclimation (2 days), and baseline (7 days), mice were singly housed, fed ad libitum, and given 24 hour running wheel access. During the scheduled food access period (14 days), mice in the STV and ABA groups had access to food only 6 hours a day from 09:00–15:00, and were removed or “dropped” from the paradigm when they lost 25% of their baseline body weight. Based on results from previous studies [36], STV mice were not expected to lose 25% baseline body weight as rapidly as ABA mice. Therefore, to induce a dropout rate equivalent to ABA mice, STV mice received a daily-adjusted, limited amount of food during the 6 hours of food access [36]. Since Ad Lib and RUN mice were also not expected to lose 25% of their baseline body weight, each Ad Lib mouse was yoked to a STV mouse, and each RUN mouse was yoked to an ABA mouse for removal from the study. Since STV mice were food restricted to drop out at the same rate as ABA mice, all four groups were removed from the study at equivalent times (see Fig 1). This design permits comparison of gene expression between groups of mice exposed to experimental conditions for the same duration. Days to dropout (loss of 25% baseline body weight) provided a measure of survival.

### RNA extraction and reverse-transcription PCR

Following dropout from the ABA paradigm, mice were sacrificed via decapitation for rapid brain extraction. Using a brain matrix and a tissue punch, the mPFC (AP 2.56 to 2.06; ML ±0.25; DV -2.00 from bregma), HPC (AP -1.56 to -2.56; ML ±1.5; DV -2.00 from bregma), NAc (AP 2.06 to 1.06; ML ±0.50; DV -5.00 from bregma), and VTA (AP -3.06 to -4.06; ML ±0.50; DV -4.50 from bregma) regions were rapidly dissected and snap frozen on dry ice. Samples were stored at -80C prior to RNA extraction. Tissue was homogenized in Qiazol lysing reagent (Qiagen, Hilden, Germany) using Lysing Matrix D tubes (MP Biomedicals, Solon, OH, USA) and the MP Biomedicals FastPrep-24 tissue ruptor (MP Biomedicals, Solon, OH, USA). RNA was extracted using the RNeasy Lipid Tissue Mini Kit (Qiagen, Hilden, Germany). Following RNA extraction, samples were analyzed for purity and RNA concentration using the Thermo Scientific NanoDrop LITE Spectrophotometer (Thermo Scientific, Waltham, MA, USA). Samples were then diluted to achieve identical RNA concentrations for reverse-transcription PCR. Reverse-transcription PCR was performed using the Taqman Reverse Transcription kit (Life Technologies, Carlsbad, CA, USA). PCR cycle is as follows: 25°C 10min, 37°C 60min, 95°C 5min.

### Quantitative real-time PCR

Samples were run in triplicate with primer/probe sets for mouse BDNF, mouse NCAM1 and eukaryotic 18S ribosomal RNA as an endogenous control (Life Technologies, Carlsbad, CA, USA) using the 7900HT Fast real-time PCR system (Life Technologies, Carlsbad, CA, USA). The PCR protocol is as follows: 50°C 2min, 95°C 10min, 95°C 15sec, 60°C 1min for 40 repeats. Samples were analyzed using the comparative Ct method for relative quantification (RQ) using the Sequence Detection Systems 2.2.1 program (Life Technologies, Carlsbad, CA, USA). An Ad Lib sample was randomly chosen as the reference control calibrator sample. Samples with a Ct standard deviation >0.25 were removed from the quantitative real-time PCR dataset. Samples with Ct values greater than 35 and/or not within 0.5 Ct of triplicate were also removed from the quantitative real-time PCR dataset.

### Statistical analysis

#### Activity-based anorexia

For baseline data, ANOVAs assessed the effects of wheel as a between-subjects factor, and day as a within-subjects factor for each dependent variable (body weight, food intake, wheel running). Posthoc ANOVAs resolved interactions of wheel x day. Bonferroni adjustments were made when posthoc ANOVAs were applied.

Mice drop out of the ABA paradigm during restriction, creating data sets with missing values. Therefore, general linear models (PROC GLIMMIX; SAS v9.2, SAS Institute, Cary, NC, USA) were used to assess differences in body weight, food intake, and wheel running during restriction. Posthoc analyses resolving wheel x day and scheduled food access x day interactions were adjusted for multiple comparisons using the false discovery rate method. Survival analysis was performed using the Kaplan-Meier test with the Logrank (Mantel-Cox) posthoc test. Mice were excluded from the entire dataset when their log transformed dropout day was > 2 SD from the group mean. Significance was set at p < .05.

#### Quantitative real-time PCR

Quantitative real-time PCR results were analyzed using the comparative Ct method for relative quantification (RQ). RQ values were log-transformed (log2(RQ)). Log-transformed RQ values > 2 SDs outside of the mean were excluded from RQ analyses. Log-transformed RQ values were compared using a two-way ANOVA with scheduled food access, and wheel access as factors. Significant interactions were resolved using post-hoc ANOVAs and/or Student-Newman-Keuls post-hoc tests. Bonferroni adjustments for number of comparisons were made when post-hoc ANOVAs are applied.

## Results

### Effects of ABA paradigm on behavior

During baseline, wheel running did not alter daily body weight (Table 1). Conversely, access to a running wheel increased food intake on baseline days 2–7 (wheel x day, F(6,26) = 4.760; p<0.0005)(all comparisons p<0.005)(data not shown). As expected, running wheel activity did not differ between mice designated to the RUN and ABA groups before scheduled feeding began.

**Table 1:** Baseline dependent measures. Body weight, food intake, and wheel running during baseline in mice housed with and without running wheels. Mean ± S.E.M.

Baseline Dependent Measures			
	Body weight (g/day)	Food intake (g/day)	Wheel running (rev/day)
Mice housed without wheels	18.980 ± 0.152	3.203 ± 0.042	33574.452 ± 1134.998
Mice housed with wheels	18.630 ± 0.126	3.957 ± 0.079	36789.429 ± 1340.382

**Fig 2:**
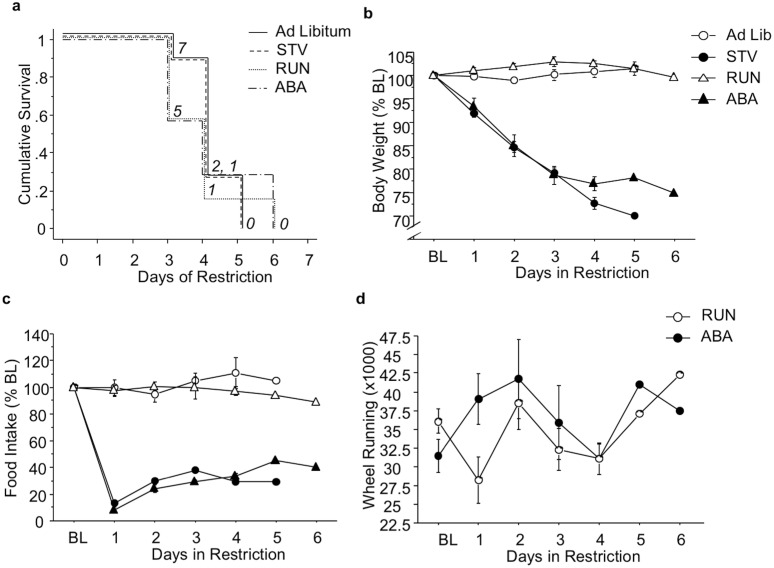
Effects of ABA paradigm on behavior. (A) Survival, (B) daily body weight, (C) food intake, and (D) wheel running during food restriction. Numbers in italics depict number of mice remaining in the ABA paradigm. Results expressed as mean ± SEM. BL, baseline; STV, starved mice exposed to food restriction and house without a wheel; RUN, mice exposed to wheel running; ABA, mice exposed to ABA conditions.

During scheduled feeding, survival did not differ between experimental groups (Fig 2A). Exposure to scheduled feeding reduced body weight on days 1–5 (restriction x day F(5,72) = 41.98; p<0.0001) (all comparisons p<0.0001) (Fig 2b). Furthermore, scheduled feeding reduced food intake on days 1–5 (restriction x day F(5,72) = 3.61; p<0.01) (all comparisons p<0.0001) (Fig 2c). Finally, scheduled feeding had no effect on wheel running activity (Fig 2d).

### Effects of ABA on gene expression

**Fig 3:**
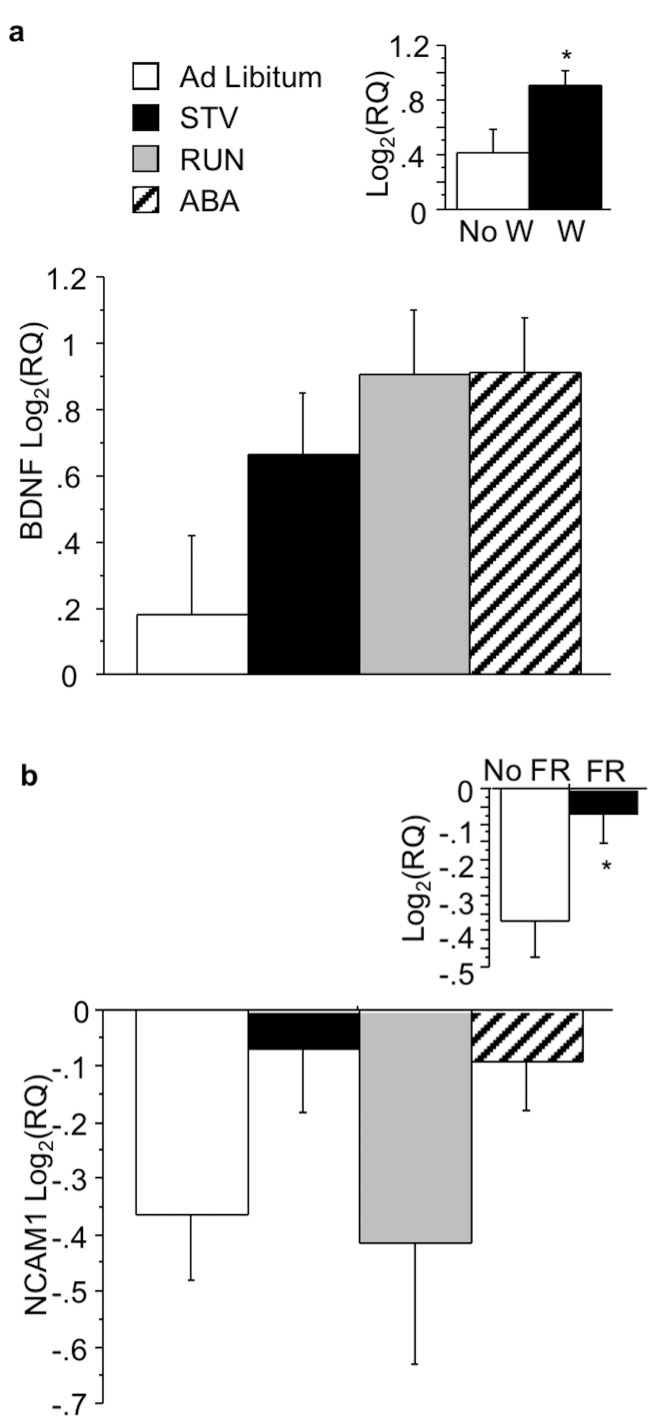
Effects of ABA on gene expression in the VTA. (A) BDNF and (B) NCAM1 expression in the VTA of mice exposed to ABA conditions. Insets indicate mean BDNF and NCAM1 expression during restriction for the independent measure depicted. Results expressed as mean log2(RQ) ± S.E.M. p<0.05. STV, starved mice exposed to food restriction and house without a wheel; RUN, mice exposed to wheel running; ABA, mice exposed to ABA conditions; W, wheel; FR, food restriction.

Within the VTA, wheel running significantly increased BDNF (F(1,22) = 4.822;p<0.05) (Fig 3A, see inset). Food restriction increased NCAM1 mRNA expression (F(1,23) = 4.537; p<0.05) (Fig 3B, see inset) in the VTA, but did not alter BDNF mRNA expression. No interactions between wheel and food access were observed.

**Fig 4:**
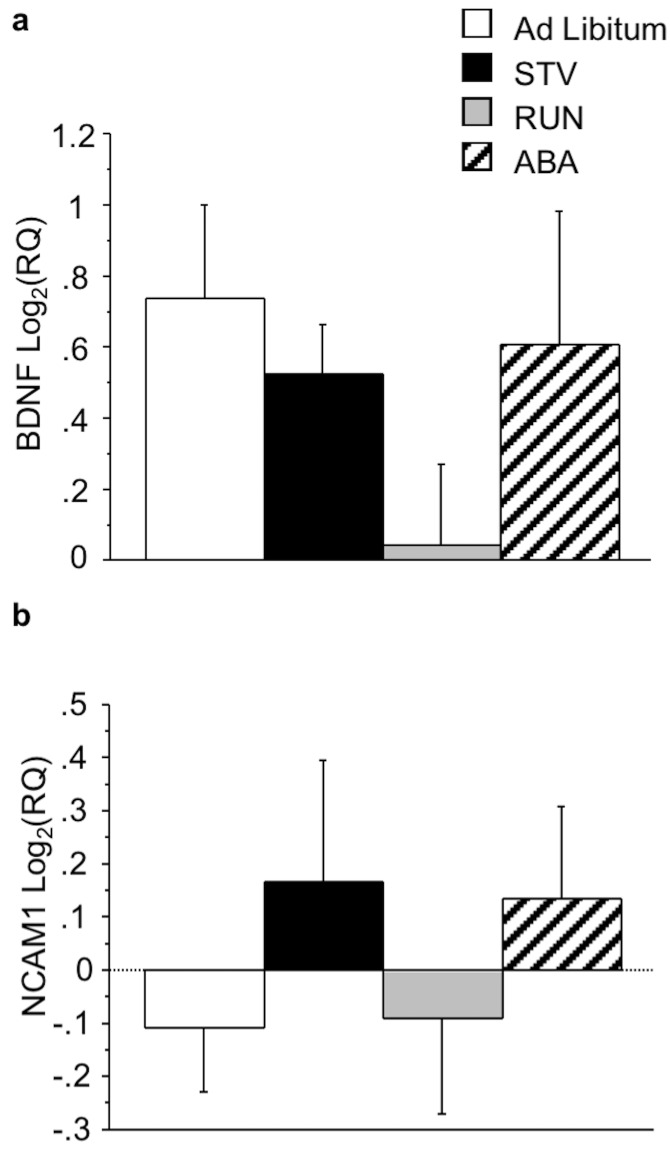
Effects of ABA on gene expression in the NAc. (A) BDNF and (B) NCAM1 expression in the NAc of mice exposed to ABA conditions. Results expressed as mean log2(RQ) ± S.E.M. STV, mice exposed to food restriction; RUN, mice with running wheel access; ABA, mice exposed to ABA conditions.

There were no main effects of wheel or food access or interactions on BDNF or NCAM1 mRNA expression within the NAc (Fig 4).

**Fig 5:**
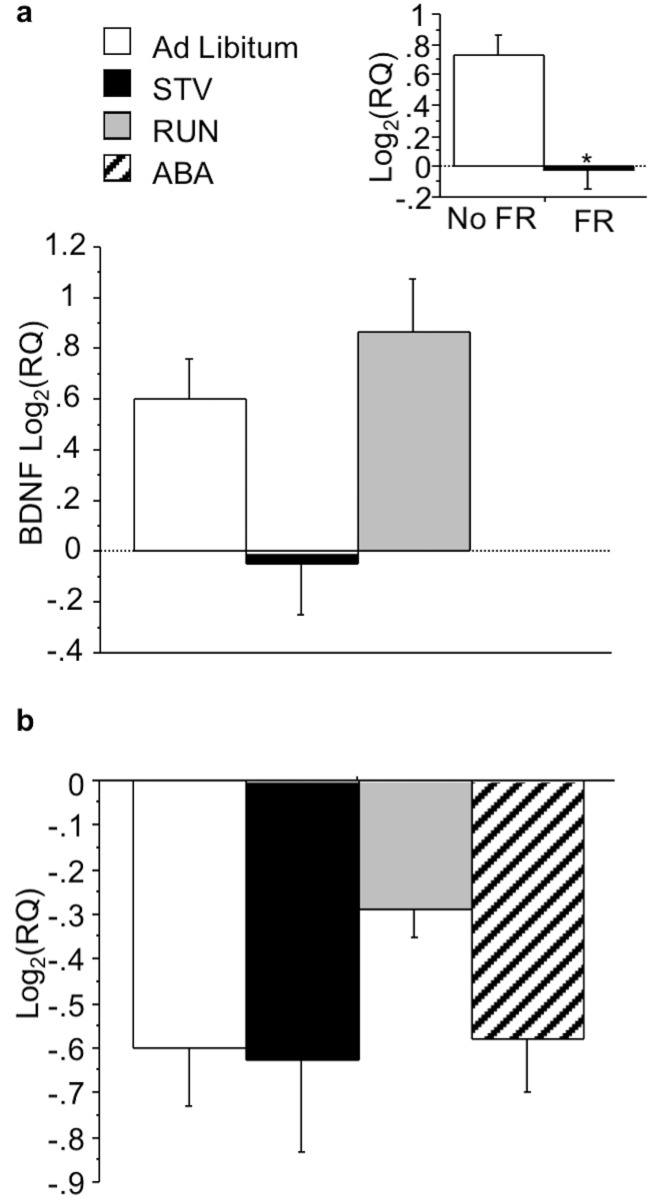
Effects of ABA on gene expression in the mPFC. (A) BDNF and (B) NCAM1 expression in the mPFC of mice exposed to ABA conditions. Inset indicates mean BDNF expression in mice subjected to food restriction or ad libitum conditions. Results expressed as mean log2(RQ) ± S.E.M. p<0.05. STV, mice exposed to food restriction; RUN, mice exposed to wheel running; ABA, mice exposed to ABA conditions; FR, food restriction.

In the mPFC, scheduled feeding decreased BDNF mRNA expression (F(1,21) = 15.366; p<0.001)(Fig 5A, see inset), but not NCAM1 mRNA expression. Wheel access did not alter BDNF or NCAM1 mRNA expression in the mPFC. No interaction of wheel access and food restriction was found.

**Fig 6:**
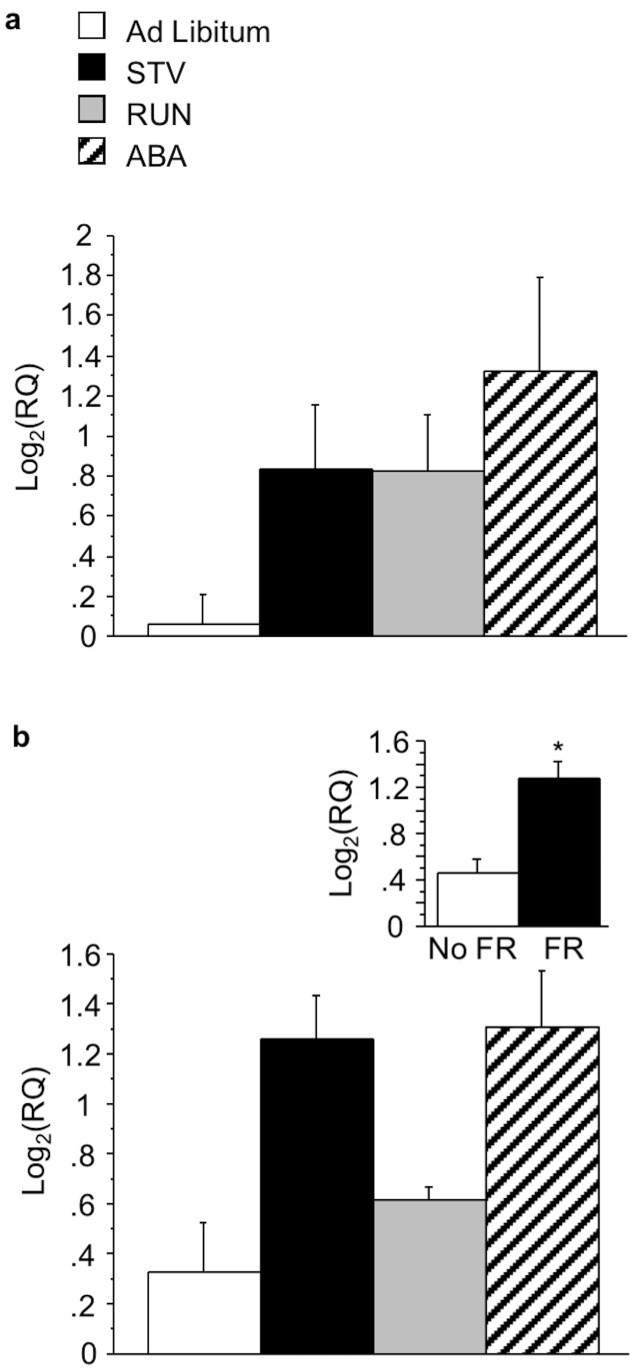
Effects of ABA on gene expression in the HPC. (A) BDNF and (B) NCAM1 expression in the HPC of mice exposed to ABA conditions. Insets indicate mean BDNF and NCAM1 expression during restriction for the independent measures depicted. Results expressed as mean log2(RQ) ± S.E.M. p<0.05. † represents a trend. STV, starved mice exposed to food restriction; RUN, mice exposed to wheel running; ABA, mice exposed to ABA conditions; W, wheel; FR, food restriction.

In the hippocampus, no effects of wheel access (F(1,23) = 3.644; p = 0.07) (Fig 6A, see inset 1) or food restriction (F(1,23) = 3.736; p = 0.07)(Fig 6A, see inset 2) were found on BDNF mRNA expression. Food restriction also increased NCAM1 mRNA expression (F(1,22) = 20.106; p<0.0005) (Fig 6B, see inset).

## Discussion

Food restriction and wheel running each induced changes in BDNF or NCAM1 mRNA expression; however, no changes in gene expression were found that were specific to the ABA condition. Food restriction led to decreases in BDNF mRNA expression in the mPFC. Furthermore, wheel running induced an increase in BDNF mRNA expression in the VTA. In addition, food restriction increased NCAM1 mRNA expression in the VTA and hippocampus, but wheel running had no effects. Finally, neither condition altered the expression of either gene in the NAc.

We generated STV and ABA groups with equivalent dropout rates by restricting the amount of chow available to STV mice. We also yoked an Ad Lib mouse to each STV mouse, and a RUN mouse to each ABA mouse, for removal from the study. Using this experimental design, we achieved similar dropout rates for the four experimental groups (Fig 1a). Importantly, there were no differences in bodyweight (Fig 1b) or food intake (Fig 1c) between Ad Lib and RUN, or between STV and ABA, groups during the study. Therefore, any potential differences in gene expression observed between ABA and STV mice could not result merely from bodyweight differences.

We found no effect of scheduled feeding on BDNF mRNA expression in the hippocampus across ABA and STV groups. Furthermore, we found no significant effect of wheel running on BDNF mRNA expression in the hippocampus across RUN and ABA groups, although we observed a trend for wheel running to increase BDNF mRNA (p = .07). Voluntary exercise has been shown to increase BDNF levels and promote neurogenesis in the hippocampus of rodents [37,38,39]. Furthermore, a previous investigation reported ABA-induced increases in BDNF mRNA expression in the hippocampus using C57Bl/6J mice [40]. Wheel running-induced increases in hippocampal BDNF levels might contribute to anorexic-like behaviors seen in the ABA paradigm.

We found that food restriction significantly decreased BDNF mRNA expression in the mPFC across ABA and STV groups. However, previous studies found no effect of food restriction on BDNF expression levels in the mPFC [41,42]. Differences in species, food restriction protocols, tissue collection, and BDNF measures may underlie the discrepancy between previous results and current findings. The mPFC is thought to modulate the salience of a given reward stimulus based on context and expectation [43]. Furthermore, BDNF and tyrosine kinase B receptor (TrkB) signaling in the mPFC has been found to regulate the consolidation of both appetitive and aversive emotional learning [44]. Direct infusion of BDNF into the mPFC normalizes drug mediated changes intracellular signaling and reward-seeking behavior in rodents [45]. Thus, food restriction-induced decreases in mPFC BDNF expression may perpetuate ABA behavior by suppressing compensatory processing of food reward.

Wheel running significantly increased BDNF mRNA expression in the VTA across ABA and RUN groups. Exercise has been previously reported to increase BDNF expression in the VTA [46]. A substantial proportion of AN patients appear to be “addicted” to exercise [47], and engage in significant physical activity daily [47]. Similarly, under ABA conditions, mice exhibit high levels of running despite very low bodyweight [48]. In support of the idea that ABA mice exhibit signs of addiction to running, mice exposed to ABA conditions show dramatic exacerbation of naloxone-induced withdrawal symptoms, indicating that ABA increases endogenous opioid levels [34]. However, viral knockdown of BDNF in the VTA enhances the rewarding effects of morphine and other opiates [49]. Therefore, increased expression of BDNF in the VTA might be predicted to reduce the expression of ABA.

We found no changes in BDNF mRNA expression in the NAc. Low baseline levels of BDNF mRNA in the NAc might have prevented detection of effects induced by the experimental conditions [50]. Although the NAc has high levels of BDNF protein, most is thought to come from anterograde transport from the VTA [51,52]. Differences in BDNF, as well as NCAM1, mRNA expression in the NAc have been reported by others for other experimental manipulations [53,54]. Experimental conditions, species, and methods implemented to quantify BDNF and NCAM1 mRNA expression may explain the disparity between previous findings and current results.

We found that NCAM1 mRNA expression was increased by food restriction in the hippocampus and in the VTA. The role of NCAM1 on feeding has not yet been well characterized. Importantly, we did not observe any brain region in which BDNF and NCAM1 transcripts were significantly altered in the same direction by either food restriction or wheel running, lending specificity to the effects of scheduled feeding and wheel access on BDNF expression.

The present study has several limitations. We assessed mRNA levels, but not protein levels, of BDNF and NCAM1. A number of studies have shown that mRNA expression levels do not always correlate with levels of protein. In addition, while we observed some changes in BDNF or NCAM1 mRNA levels in response to scheduled feeding or wheel running, we did not observe any interactions between these conditions on gene expression. Although the effects of wheel running and scheduled feeding on BDNF mRNA levels do not interact within one brain structure, these factors may influence ABA through their actions within the same neural circuit.

The results of the present study identify BDNF expression within mesocorticolimbic brain structures as a reasonable target for further investigations into the neural mechanisms underlying ABA. We identified changes in BDNF mRNA expression within mesocorticolimbic structures that were induced by food restriction or wheel access. These findings contribute to our scant knowledge of the molecular underpinnings of ABA. Further studies are underway to identify whether manipulation of BDNF expression mediates the hypophagia or hyperactivity observed in ABA. Such work will be critical for understanding the etiology of anorexia nervosa and developing treatments for this disorder.

## Supporting Information

S1 File ABA paradigm daily measures. Excel spreadsheet including raw daily measurements for body weight, food intake, and running wheel activity throughout the ABA paradigm.
(XLSX)

S2 File VTA qPCR data. Excel spreadsheet including raw data output from qPCR plates run with VTA samples.
(XLSX)

S3 File NAc qPCR data. Excel spreadsheet including raw data output from qPCR plates run with NAc samples.
(XLSX)

S4 File mPFC qPCR data. Excel spreadsheet including raw data output from qPCR plates run with mPFC samples.
(XLSX)

S5 File HPC qPCR data. Excel spreadsheet including raw data output from qPCR plates run with HPC samples.
(XLSX)
